# Enzymatic liver function measured by LiMAx is superior to current standard methods in predicting transplant-free survival after TIPS implantation

**DOI:** 10.1038/s41598-021-93392-5

**Published:** 2021-07-05

**Authors:** Jassin Rashidi-Alavijeh, Alisan Kahraman, Guido Gerken, Jens M. Theysohn, Katharina Willuweit, Dieter P. Hoyer, Christian M. Lange, Matthias Buechter

**Affiliations:** 1 Department of Gastroenterology and Hepatology, University Hospital Essen, University of Duisburg-Essen, Essen, Germany; 2 Department of Diagnostic and Interventional Radiology and Neuroradiology, University Hospital Essen, University of Duisburg-Essen, Essen, Germany; 3 Department of General-, Visceral- and Transplantation Surgery, University Hospital Essen, University of Duisburg-Essen, Essen, Germany; 4 St. Nikolaus-Stiftshospital, Ernestus-Platz 1, 56626 Andernach, Germany

**Keywords:** Liver cirrhosis, Portal hypertension

## Abstract

Transjugular intrahepatic portosystemic shunt (TIPS) is one of the main treatment options in patients with decompensated liver cirrhosis but is still associated with partly severe complications. For adequate patient selection, prognostic parameters are of crucial importance. The liver maximum capacity (LiMAx) breath test measures enzymatic liver function and could potentially represent an efficient prognostic marker. We therefore aimed to assess the role of LiMAx in predicting survival of TIPS patients in a prospective analysis. LiMAx was performed for patients who underwent TIPS implantation between October 2016 and February 2018. Associations with transplant-free survival after 24 weeks were assessed by logistic regression. A total number of 30 patients were included, of whom seven received liver transplantation (N = 2) or died (N = 5) during follow-up. LiMAx values after (*P* = 0.01, OR = 1.24, 95% CI = 1.04–1.47) and before (*P* = 0.03, OR 1.21, 95% CI = 1.02–1.43) TIPS implantation and MELD score (*P* = 0.03, OR = 0.79, 95% CI = 0.63–0.98) were significantly associated with transplant-free survival according to univariate logistic regression. In AUROC analysis, LiMAx at day one after TIPS (sensitivity 85.7%, specificity 78.3%, AUROC 0.85, cut-off ≤ 165 µg/kg/h), LiMAx value at the day before TIPS (sensitivity 100%, specificity 73.9%, AUROC 0.82, cut-off ≤ 205 µg/kg/h) and MELD score (sensitivity 71.4%, specificity 73.9%, AUROC 0.82, cut-off ≥ 15) had the highest prognostic accuracy. LiMAx values prior and after TIPS procedure seem to be good prognostic parameters regarding prediction of transplant-free survival of patients undergoing TIPS implantation.

## Introduction

Portal hypertension (PH) is one of the main reasons for morbidity and mortality among patients with liver cirrhosis and is defined by a hepatic venous pressure gradient (HVPG) ≥ 6 mmHg. Still, PH normally starts to develop clinical relevance when exceeding the threshold of 10 mmHg with risk of ascites and esophageal varices, thereby defined as clinically significant portal hypertension (CSPH)^1–3^. Upon the occurrence of ascites and/or variceal bleeding, which is then called decompensated portal hypertension (DPH), mortality is escalating rapidly^4^.

In case of DPH, the placement of transjugular intrahepatic portosystemic shunt (TIPS) is an important treatment option, particularly regarding the therapy of recurrent hydropic decompensation^5–8^ and/or variceal bleeding events^9–13^. However, implantation of TIPS is associated with different complications, such as occurrence of cardiac decompensation^14^, hepatic encephalopathy (HE)^15–18^ or impairment of liver function^19–21^, which can even lead to subsequent liver failure, being the most important of them.

For these reasons, an adequate selection of patients receiving TIPS is of crucial importance. Advanced age, elevated MELD score or bilirubin levels, decreased number of platelets and previous episodes of HE are known risk factors and probable contraindications of TIPS placement^21–26^. However, although these risk factors have been partially known for years, the number of complications after TIPS is still significant, suggesting the necessity of new prognostic markers.

The liver maximum capacity (LiMAx) breath test is a novel non-invasive quantitative test to measure enzymatic liver function by determining hepatic metabolization of ^13^C-labeled methacetin by cytochrome P450 1A2 and was initially evaluated to assess operability of patients undergoing hepatic surgery^27–29^. Still, it was also successfully evaluated for other clinical situations, e. g. estimation of survival of patients with acute liver failure^30^ or liver transplant (LT) candidates^31^ and for prediction of hepatic disease severity/grade of liver fibrosis^32,33^.

Currently, Reichert et al*.* prospectively analyzed the predictive power of LiMAx in TIPS patients and stated that the drop of LiMAx after TIPS is associated with occurrence of liver function-associated complications, including occurrence of HE and liver failure^34^. However, impact of LiMAx results on survival of TIPS patients has not been reported. To the best of our knowledge, other studies regarding prediction of TIPS outcome by LiMAx have not been contributed thus far. Hence, we performed a prospective study to assess the predictive power of LiMAx on transplant-free survival of patients after TIPS placement.

## Patients and methods

### Study population

**Figure 1:**
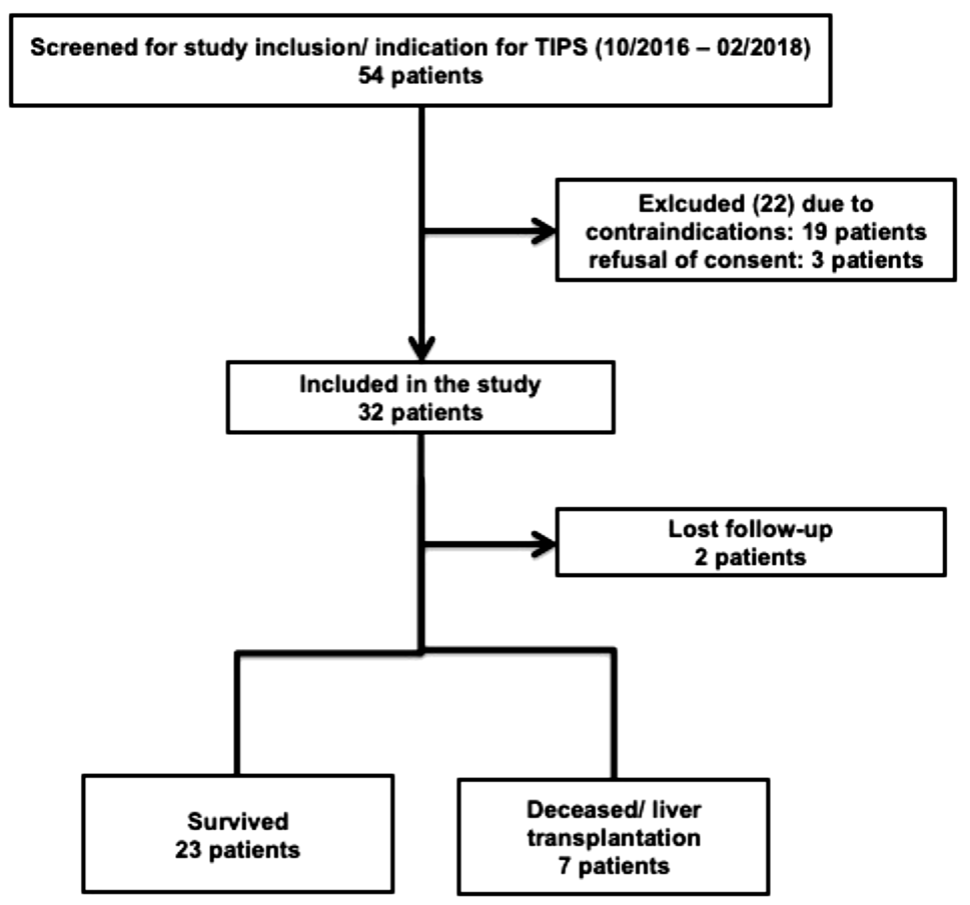
Patient selection.

The ethics committee of the University Hospital Essen approved this prospective monocentric observational study (16-7228-BO). The study was conducted in accordance with the Declaration of Helsinki. Written informed consent was obtained from each patient at inclusion. All patients evaluated for TIPS implantation at the Department of Gastroenterology and Hepatology at the University Hospital Essen between October 2016 and February 2018 were screened for study inclusion. Only patients suffering from decompensated cirrhotic portal hypertension who were eligible for elective TIPS implantation without having contraindications according to current recommendations^2,35^ were included. In addition, age under 18 years, paracetamol allergy, and the inability to take a part in the study examinations were defined as exclusion criteria. Patients were followed up for 24 weeks after TIPS implantation. Primary endpoints were defined as follows: (i) death or liver transplantation within 24 weeks or (ii) presentation at 24-week follow-up examination. A flowchart regarding patient selection is demonstrated in Fig. 1.

### Angiographic measurement and TIPS placement

After puncturing the right internal jugular vein under local anaesthesia and ultrasound guidance, a guide wire (Merit Medical) was positioned in the inferior vena cava. A 10F introducer sheath (Gore, Germany) was then inserted. To gain access to the right hepatic vein, a 5F multi-purpose catheter (Cordis) was used. The sheath was advanced into the hepatic vein and a balloon catheter was wedged to obtain an indirect portogram. To calculate HVPG, measurements of free and wedged hepatic venous pressure were performed. The portal vein was punctured ultrasound-guided and under fluoroscopic control using a 16 Gauge needle (Gore). Then, a stiff guide wire (Amplatz Superstiff Wire, Boston Scientific, Natick, MA, USA) was advanced into the portal venous system and a pig-tail catheter (Cordis) was placed into the main portal vein to first measure the direct portal pressure and second to perform a direct portogram. Hereafter, the portovenous PTFE covered stent (Viatorr, Gore) was implanted under fluoroscopic control and dilated to widths between 6 and 10 mm using an angioplasty balloon (Boston Scientific, Natick, MA, USA) to reach a HVPG < 13 mmHg. Pressure gradients between the portal vein and the inferior vena cava were measured before dilatation, after establishing the shunt and after each additional dilatation with a wider balloon.

### LiMAx measurement

The LiMAx test (Humedics, Berlin, Germany) was performed after a minimum of 3 h fasting. The measurement is based on the hepatocellular-specific metabolism of intravenously administered ^13^C-labeled methacetin—an exclusive substrate for the hepatic cytochrome P450 1A2 enzyme. In hepatocytes, ^13^C-methacetin is immediately demethylated into acetaminophen and ^13^CO_2_; the latter is subsequently exhaled, leading to an increase of ^13^CO_2_ concentration in expiration. Prior to substrate injection, patient’s individual baseline ratio ^13^CO_2_/^12^CO_2_ concentration is measured and thus liver function capacity can be calculated from the analysis of the ^13^CO_2_/^12^CO_2_ ratio within 60 min after injection. Results are given in µg/kg/h. LiMAx measurements were performed on the following time points: (i) one day before TIPS implantation, (ii) one day after TIPS implantation, (iii) four weeks after TIPS implantation, and (iv) 12 weeks after TIPS implantation, respectively.

### Statistical analysis

The results presented are based on exploratory analyses planned after study completion and should be interpreted accordingly. Data were used as available where no missing data occurred except for LiMAx measurements at week 4 and 12 after TIPS implantation if the patient deceased/had LT before the assessment. Percentages for baseline and demographic characteristics are related to the number of patients in the respective group. Corresponding comparisons between survivors and deceased patients / patients with LT were either done using a two-sided non-parametric Mann–Whitney U-test in case of continuous / ordinal data or using a two-sided Fisher’s exact test in case of categorical data. In case of more than two categories, the extended version of Fisher’s exact test by Freeman and Halton was used. The univariate logistic regression models with logit link function used to obtain estimates related to odds ratios and receiver operating curves included outcome (survivor vs. death / LT) as dependent variable and one (possible) prognostic factor as independent variable. The area under the receiver operation curve (AUROC) is used as measure for the prediction performance of the models. Decisions about the optimal cut-off value are based on the Youden index defined as sum of sensitivity and specificity minus one. For all results, a p-value lower 0.05 is considered statistically significant. Statistical analysis was performed using SAS version 9.4 (SAS Institute Inc., Cary, North Carolina, USA).

## Results

### Patient characteristics

A total of 30 patients were included in this prospective study. Median patient age was 55.5 years (range, 38–74) and 67% of patients were male. The etiology of liver cirrhosis was alcoholic in most cases (73%), followed by non-alcoholic steatohepatitis (NASH)-related cirrhosis (17%), hepatitis C virus (HCV, 3%), primary biliary cholangitis (PBC, 3%) and autoimmune hepatitis (AIH, 3%). The indication for TIPS placement was refractory ascites in 83% of patients and recurrent variceal bleeding in 17%. Most patients presented with Child Pugh stage B (67%), while 10% presented stage A and 23% stage C. None of the patients included in this study received “rescue TIPS” due to uncontrollable variceal hemorrhage. Seven of these patients died (n = 5) or received LT (n = 2) during follow-up, while 23 survived without LT. Both patients receiving LT showed significant impairment of liver function after TIPS implantation with relevant increase of bilirubin levels and MELD score (18 and 20 at time of LT, respectively) and were therefore considered for LT. All patients who died showed severe deterioration of liver function, leading to multiorgan failure including hepatic encephalopathy. Two patients died after severe pneumonia (infection with influenza in one case and with pneumocystis jirovecii in the other), one showed decompensation after spontaneous bacterial peritonitis, and the other two patients developed rapid reduction of liver function and multiorgan failure without verification of a specific cause, presumably due to reduced hepatic reserve after TIPS implantation. Median time to death/LT was 56 days after TIPS implantation. More detailed demographic and clinical data are presented in Table 1.

**Table 1:** Baseline and demographic characteristics.

	Total (n = 30)	Survivors (n = 23)	Death/LT (n = 7)	*P*-value
Male sex, n (%)	20 (67)	14 (61)	6 (86)	0.37
Age (years), median (range)	55.5 (38–74)	54 (38–71)	62 (47–74)	0.22
LiMAx one day before TIPS [µg/kg/h], median (range)	233 (77–538)	249 (77–538)	178 (90–205)	**0.01**
LiMAx one day after TIPS [µg/kg/h], median (range)	195 (65–345)	207 (65–345)	80 (75–214)	** < 0.01**
LiMAx drop one day after TIPS [µg/kg/h], median (range)	-44.5 (-303–58)	-44 (-303–58)	-46 (-98–34)	0.86
HVPG pre TIPS [mmHg], median (range)	22 (14–34)	23 (15–34)	22 (14–29)	0.31
HVPG post TIPS [mmHg], median (range)	9 (5–12)	9 (5–12)	8 (5–12)	0.44
HVPG drop [mmHg], median (range)	14 (6–24)	14 (6–24)	13 (7–17)	0.54
TIPS diameter [mm], median (range)	8 (6–10)	8 (6–10)	6 (6–8)	0.14
MELD score, median (range)	12 (6–23)	10 (6–22)	16 (10–23)	**0.02**
Child Pugh score [points], median (range)	8 (5–11)	8 (5–11)	9 (7–11)	0.05
**Child Pugh stage**				0.40
Class A, n (%)	3 (10)	3 (13)	0 (0)	
Class B, n (%)	20 (67)	16 (70)	4 (57)	
Class C, n (%)	7 (23)	4 (17)	3 (43)	
**Origin of cirrhosis (n, %)**				0.60
Alcohol	22 (73)	17 (74)	5 (71)	
NASH	5 (17)	4 (17)	1 (14)	
HCV	1 (3)	0 (0)	1 (14)	
PBC	1 (3)	1 (4)	0 (0)	
AIH	1 (3)	1 (4)	0 (0)	
**Indication of TIPS placement (n, %)**				0.30
Ascites	25 (83)	18 (78)	7 (100)	
Variceal bleeding	5 (17)	5 (22)	0 (0)	
**Laboratory parameters (median, SD)**				
Platelets (per nL)	145 (124.1)	151 (134.9)	103 (49.9)	**0.04**
Creatinine (mg/dL)	1.1 (1.3)	1.1 (1.5)	1.9 (0.6)	0.11
Bilirubin (mg/dL)	1.0 (1.0)	1.0 (0.9)	1.8 (1.1)	0.29
AST (U/L)	41 (33.7)	42 (37.8)	36 (14.8)	0.35
ALT (U/L)	21 (20.4)	21 (23.4)	21 (6.7)	0.92
Albumin (g/dL)	3.2 (0.6)	3.2 (0.7)	3.0 (0.4)	0.59
INR	1.16 (0.2)	1.12 (0.2)	1.34 (0.2)	0.07

Bold values indicate statistically significant at *P* < 0.05.

*AIH* autoimmune hepatitis, *ALT* alanine aminotransferase, *AST* aspartate aminotransferase, *HCV* hepatitis C virus, *HVPG* hepatic venous pressure gradient, *INR* international normalized ratio, *MELD* model for end-stage liver disease, *NASH* non-alcoholic steatohepatitis, *PBC* primary biliary cholangitis, *TIPS* transjugular intrahepatic portosystemic shunt.

### TIPS implantation leads to significant impairment of liver function partly recovering in the course

**Figure 2:**
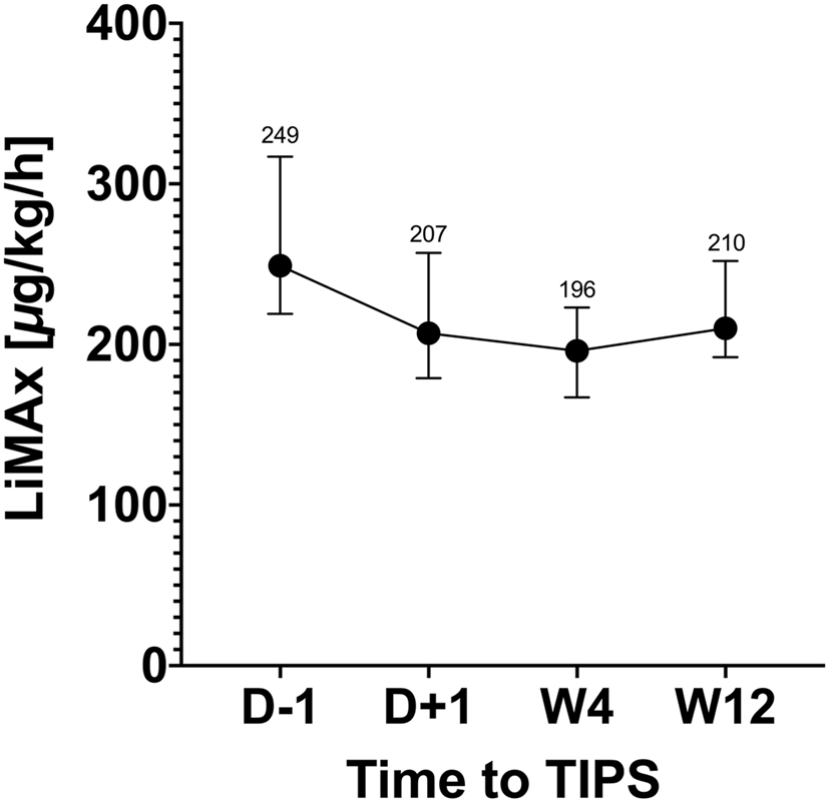
Course of LiMAx values related to TIPS implantation among survivors (N = 23), *P* = 0.012 (D-1: one day before TIPS; D + 1: one day after TIPS; W4: 4 weeks after TIPS; W12: 12 weeks after TIPS).

**Figure 3:**
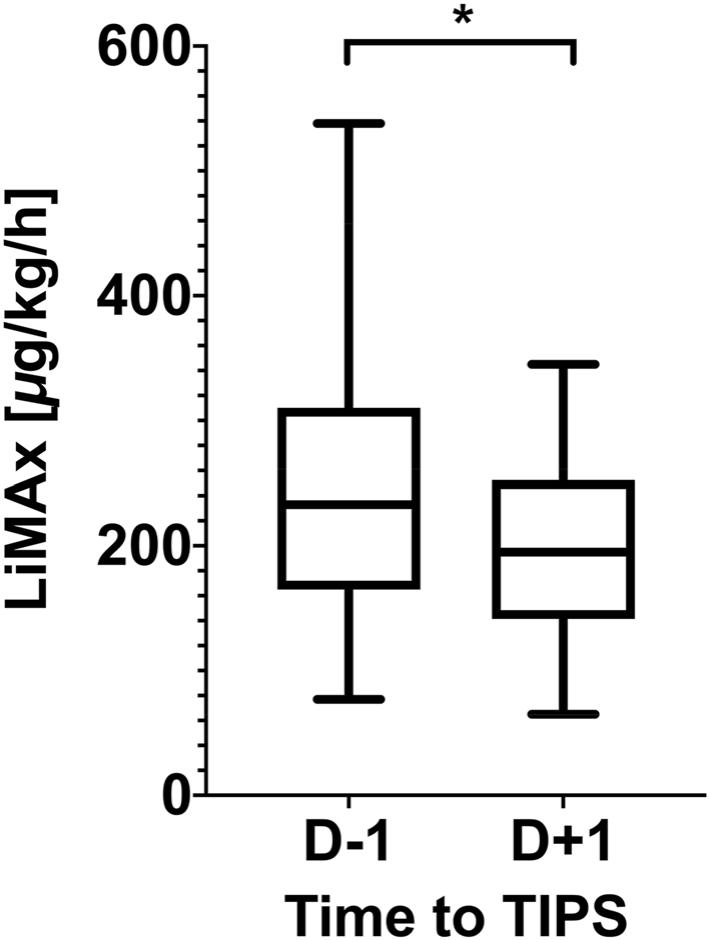
TIPS implantation leads to significant decrease of LiMAx values (N = 30, *P* = 0.03). (D − 1: one day before TIPS; D + 1: one day after TIPS).

Median LiMAx values were significantly lower in the group who died or received LT one day before TIPS (178 µg/kg/h) and one day after TIPS (80 µg/kg/h) compared to those who survived without LT (249 µg/kg/h and 207 µg/kg/h, respectively). Additionally, MELD score was significantly higher (16 vs. 10) and platelet count was significantly lower (103/nL vs. 151/nL) in the death/LT group compared to those who survived without LT (Table 1). After TIPS implantation, LiMAx values decreased significantly (Fig. 2, *P* = 0.03), but resolved partly after three months in those patients who survived, although they did not reach baseline levels (Fig. 3). However, a potential correlation between LiMAx and HVPG was not found (R = 0.058, *P* = 0.75). In addition, we evaluated correlation coefficients for LiMAx and Child Pugh score, MELD score and the novel Freiburg index of post-TIPS survival (FIPS){Bettinger, 2021 #56} (Bettinger et al. 2020, Journal of Hepatology). We found low correlation for LiMAx and MELD score (R = -0.38, P = 0.04) and moderate correlation for LiMAx/Child Pugh score (R = −0.55, *P* = 0.001) and LiMAx/FIPS (R = -0.50, *P* = 0.001), respectively.

### Analysis of different parameters as predictors of transplant-free survival

We analyzed different parameters and, in particular, different LiMAx values before and after TIPS implantation for predicting transplant-free survival. In univariate analysis, LiMAx value at day after TIPS (*P* = 0.01, OR = 1.24, 95% CI = 1.04–1.47), MELD score (*P* = 0.03, OR = 0.79, 95% CI = 0.63–0.98) and LiMAx value at day before TIPS (*P* = 0.03, OR 1.21, 95% CI = 1.02–1.43) showed significant association with transplant-free survival. Since all three parameters are dependent and correlate with each other, multivariate analysis was not considered conducive and was therefore not performed. Association between different prognostic parameters and transplant-free survival after TIPS is demonstrated in Table 2.

**Table 2:** Univariate analysis of association between different possible prognostic parameters and transplant-free survival after TIPS.

	Univariate analysis	
	OR (95% CI)	*P*-value
Limax value at day after TIPS	1.24 (1.04–1.47)	**0.01**
MELD score	0.79 (0.63–0.98)	**0.03**
LiMAx one day before TIPS	1.21 (1.02–1.43)	**0.03**
INR	0.62 (0.37–1.03)	0.06
Child Pugh score	0.51 (0.24–1.06)	0.07
Quick	1.06 (0.99–1.13)	0.08
Platelets	1.02 (1.00–1.03)	0.09
Age	0.94 (0.85–1.03)	0.18
Bilirubin (mg/dL)	0.95 (0.87–1.03)	0.21
AST (U/L)	1.02 (0.98–1.08)	0.32
HVPG pre TIPS	1.10 (0.91–1.33)	0.33
HVPG post TIPS	1.18 (0.79–1.75)	0.42
HVPG drop	1.08 (0.88–1.33)	0.46
ALT (U/L)	1.02 (0.96–1.09)	0.53
Albumin (g/dL)	1.04 (0.90–1.20)	0.60
LiMAx drop one day after TIPS	0.97 (0.87–1.09)	0.62
Creatinine (mg/dL)	0.99 (0.93–1.05)	0.67

Bold values indicate statistically significant at *P* < 0.05.

*ALT* alanine aminotransferase, *AST* aspartate aminotransferase, *HVPG* hepatic venous pressure gradient, *INR* international normalized ratio, *MELD* model for end-stage liver disease, *TIPS* transjugular intrahepatic portosystemic shunt.

### Analysis of diagnostic accuracy of different parameters in prediction of transplant-free survival after TIPS

**Figure 4:**
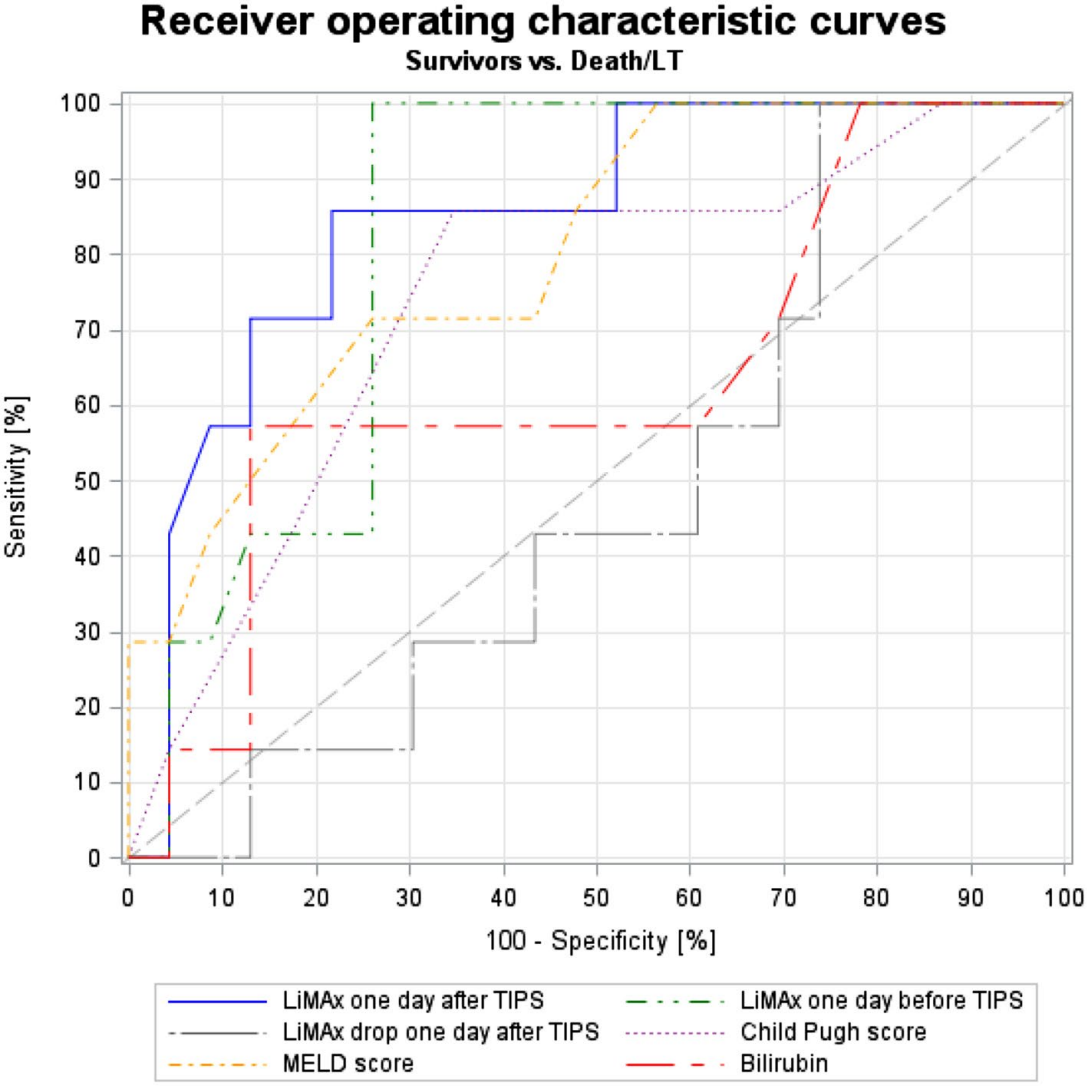
Receiver operating characteristics (ROC) curves of five parameters with highest AUROC values and LiMAx drop in prediction of transplant-free survival after TIPS implantation.

For detection of diagnostic accuracy of different parameters, AUROC analysis was performed. In AUROC analysis, LiMAx value at day after TIPS had the highest diagnostic accuracy in predicting transplant-free survival (sensitivity 85.7%, specificity 78.3%, AUROC 0.85, cut-off ≤ 165 µg/kg/h), followed by LiMAx value at day before TIPS (sensitivity 100%, specificity 73.9%, AUROC 0.82, cut-off ≤ 205 µg/kg/h) and MELD score (sensitivity 71.4%, specificity 73.9%, AUROC 0.82, cut-off ≥ 15). Interestingly, total bilirubin, which is often used for prediction of TIPS outcome, did not perform well (sensitivity 57.1%, specificity 87.0%, AUROC 0.63, ≥ 1.8 mg/dl), neither did LiMAx drop at day after TIPS (sensitivity 100%, specificity 26.1%, AUROC 0.49, ≥ -98). ROC curves of five parameters with highest AUROC values and LiMAx drop are demonstrated in Fig. 4. More detailed data regarding AUROC analysis, including positive predictive values, negative predictive values and Youden-index, are presented in Table 3.

**Table 3:** Analysis of diagnostic accuracy of different parameters in prediction of transplant-free survival after TIPS.

	Sensitivity [%]	Specificity [%]	PPV [%]	NPV [%]	Cut-off	Youden index	AUROC
LiMAx one day before TIPS	100.0	73.9	53.9	100.0	≤ 205	0.74	0.82
LiMAx one day after TIPS	85.7	78.3	54.6	94.7	≤ 165	0.64	0.85
Creatinine	71.4	87.0	62.5	90.9	≥ 1.87	0.58	0.70
Child Pugh points	85.7	65.2	42.9	93.8	≥ 9	0.51	0.74
MELD score	71.4	73.9	45.5	89.5	≥ 15	0.45	0.80
INR	57.1	87.0	57.1	87.0	≥ 1.34	0.44	0.73
Bilirubin	57.1	87.0	57.1	87.0	≥ 1.8	0.44	0.63
Platelets	100.0	43.5	35.0	100.0	≥ 165	0.43	0.76
Age	71.71.43	69.6	41.7	88.9	≥ 61	0.42	0.65
LiMAx drop at day after TIPS	100.0	26.1	29.2	100.0	≥ -98	0.26	0.49
AST	42.9	82.6	42.9	82.6	≤ 29	0.25	0.62
Albumin	85.7	39.1	30.0	90.0	≤ 3.4	0.25	0.57
HVPG before TIPS	71.4	52.2	31.3	85.7	≤ 22	0.24	0.63
Change in HVPG	100.0	21.7	28.0	100.0	≤ 17	0.22	0.58
HVPG after TIPS	42.9	78.3	37.5	81.8	≤ 6	0.21	0.60
ALT	100.0	17.4	26.9	100.0	≤ 33	0.17	0.49

*ALT* alanine aminotransferase, *AST* aspartate aminotransferase, *AUROC* area under receiver operating characteristics, *HVPG* hepatic venous pressure gradient, *INR* international normalized ratio, *MELD* model for end-stage liver disease, *NPV* negative predictive value, *PPV* positive predictive value, *TIPS* transjugular intrahepatic portosystemic shunt.

## Discussion

Decompensated portal hypertension (DPH), defined by the occurrence of ascites and/or variceal bleeding, is the main factor determining prognosis in patients with liver cirrhosis^4^, and TIPS implantation is one of the most established therapeutic options to reduce complications of portal hypertension. Although TIPS can significantly reduce morbidity and in part mortality, the procedure has relevant adverse effects^15,16,19^, therefore selection of patients being suitable for TIPS implantation is of crucial importance. Impairment of liver function seems to be the main predicting factor for complications after TIPS implantation, and different parameters which could possibly forecast liver failure and consecutively TIPS outcome have been proposed^21–25^. However, these parameters do not seem to be reliable enough, since TIPS-associated complications are still a relevant problem among this patient collective. Thus, identification of a reliable prognostic tool will have the benefit of clearer patient selection, thereby avoiding high numbers of TIPS-associated adverse events and, finally, decline of patients who could profit from TIPS implantation.

Different studies have shown that the LiMAx breath test is able to determine enzymatic liver function and therefore can effectively predict outcome in various clinical settings, like for patients undergoing hepatic surgery or patients with acute liver failure^27–30^. In addition, we could demonstrate strong correlation between LiMAx and the degree of chronic liver disease, cirrhosis and portal hypertension in previous studies of our group^32,33^. Hence, we evaluated the ability of LiMAx to predict outcome after TIPS placement, in comparison to other, more established parameters.

Regarding literature, studies analyzing the association between LiMAx testing and TIPS implantation are scarce. There is only one prospective study conducted by Reichert et al*.*, in which the drop of LiMAx after TIPS showed significant association with liver-function associated complications, like HE and early liver failure^34^. Still, there are no reports on prediction of transplant-free survival after TIPS implantation by LiMAx testing.

In the present study, enzymatic liver function measured by LiMAx at day before TIPS (*P* = 0.03), at day after TIPS (*P* = 0.01) and MELD score (*P* = 0.03) showed significant association with transplant-free survival. Additionally, in AUROC analysis, it was the same three parameters showing highest diagnostic accuracy in predicting outcome.

While the role of MELD score in predicting patients’ outcome after TIPS implantation is already reported previously and well established as a possible risk factor of this intervention^23,36,37^, our findings regarding LiMAx testing prior and after TIPS placement present a new aspect and offer relevant additional information. Hence, we believe that LiMAx could be a beneficial tool for determining patient outcomes for TIPS candidates in the future. In our cohort, the LiMAx value at day before TIPS was a significant predictor of outcome (*P* = 0.03) and had high diagnostic accuracy (sensitivity 100%, specificity 73.9%, AUROC 0.82, cut-off ≤ 205 µg/kg/h). These results demonstrate that LiMAx testing before TIPS can play an important role in adequate patient selection and improving individual prediction of outcome. Of course, further validation of our cut-off value is necessary, and clearly, other components, like MELD score, bilirubin levels and Child Pugh stage, will still play an important role in patient selection and outcome. Nevertheless, our results demonstrate that pre-interventional assessment of liver function with the LiMAx test might constitute a decisive component in future perspectives.

In addition, LiMAx value at day after TIPS was significantly influencing outcome (*P* = 0.01), and showed highest diagnostic accuracy in predicting transplant-free survival (sensitivity 85.7%, specificity 78.3%, AUROC 0.85, cut-off ≤ 165 µg/kg/h). Post-interventional assessment of liver function by LiMAx test might therefore help preventing severe adverse events such as liver failure by a more extensive and individual surveillance in patients with LiMAx values below cut-off.

In the study of Reichert et al*.*, LiMAx drop at day after TIPS performed very well in predicting liver-associated complications. However, in our study, LiMAx drop did not show significant results regarding outcome after TIPS implantation, but it has to be considered that the endpoint of our study differed from that of Reichert et al*.* (transplant-free survival vs. liver-associated complications).

Although we did not explicitly analyze liver associated complications after TIPS-implantation, all patients receiving LT and all patients who died suffered from liver-associated complications, and except of these, only two other patients had distinct liver-associated complications (hepatic encephalopathy grade 2). Therefore, a high correlation between transplant-free survival and liver-associated complications can be expected. Since both the study of Reichert et al*.* and our study were designed as pilot studies with rather small patient collectives, further prospective studies with larger cohorts should be performed to clarify these aspects.

We furthermore hypothesized that there could be a connection between the extent of portal hypertension, measured by HVPG, and enzymatic liver function measured by LiMAx, meaning the higher the HVPG the lower the LiMAx. However, this negative correlation could not be confirmed among our cohort.

In principle, LiMAx depends upon functional enzymatic liver reserve, but should also be influenced by blood flow/liver perfusion. It is therefore plausible that bypassing blood flow via TIPS and decreasing HVPG should correlate with LiMAx drop. In case of TIPS implantation, correlation between drop of LiMAx and HVPG would have been expected, since functional enzymatic liver reserve should not be altered by TIPS implantation immediately. But actually, decrease of LiMAx was not as pronounced as decrease of HVPG (20% vs. 59%, respectively).

This lack of linear correlation could have different reasons. One possible and plausible explanation might be that portal pressure, as measured by HVPG, does not inevitably correlate with blood volume perfusing the liver. In state of cirrhosis, liver resistance is high, thereby inducing high pressure in the visceral blood circulation, leading to varices in different organ systems. After TIPS implantation, portal pressure is rapidly decreasing by inserting a bypass, which can easily be recognized by reduction of HVPG. In addition, total blood volume perfusing the liver should not decline as fast as HVPG, since loss of resistance in the liver leads to mobilization of large blood volume from other organ systems, especially from esophageal and gastric varices, which can consecutively be documented by immediate disappearance of these varices. Furthermore, neurohumoral and hemodynamic changes after reducing portal pressure (e.g., vasodilatation in splanchnic system) lead to increase of blood flow in the hepatic artery. For these reasons, both LiMAx and HVPG decrease, but LiMAx does not drop as fast and pronounced as does HVPG. However, analysis and correlation between LiMAx and blood perfusion before and after TIPS implantation will perform better in this regard and might be an interesting issue for future perspectives.

There are other parameters, which were declared as risk factors of TIPS implantation before, like elevated bilirubin levels and Child Pugh stage^21,23,24,38–41^. Some of these factors were analyzed in our study, too, but did not perform well. Neither total bilirubin levels (*P* = 0.21)—which might be the most established parameter for patient selection –, nor Child Pugh stage (*P* = 0.4), HVPG drop (*P* = 0.54) and age (*P* = 0.22) did show significant results regarding outcome after TIPS implantation. However, the relatively small cohort size might be an explanation for these findings, as well being that patients were carefully selected in consideration of established parameters, thereby excluding most patients with higher Child Pugh score or elevated bilirubin levels, leading to restricted variance of our parameters and thereby limiting possible significance of their predictive value. According to actual recommendations regarding bilirubin levels, for example, we only included two patients with bilirubin levels > 3 mg/dl (and none with bilirubin levels > 5 mg/dl), and for this reason, it is not surprising that these parameters miss to yield statistical significance. However, it is remarkable that LiMAx values were still significant despite exclusion of patients with strongly impaired hepatic function and determined prognosis accurately in AUROC analysis—indicating high specificity of LiMAx test regarding restriction on liver function.

We are aware of the limitations of our study, the most important of them being the relatively small cohort size and being a single-center study. We still believe that these prospective data are of high clinical importance, since they emphasize the predictive value of LiMAx testing prior and after TIPS implantation, thereby showing that assessment of liver function by LiMAx can be a powerful additional tool for clinicians regarding individual patient selection and post-interventional surveillance. However, larger, multi-center studies are warranted for confirming of these results.

### Ethical approval

 The ethics committee of the University Hospital Essen approved this prospective study (16–7228-BO). The study was conducted in accordance with the Declaration of Helsinki.

### Consent to publish

Written informed consent was obtained from each patient before inclusion.

## Abbreviations

PH: Portal hypertension
HVPG: Hepatic venous pressure gradient
CSPH: Clinically significant portal hypertension
DPH: Decompensated portal hypertension
TIPS: Transjugular intrahepatic portosystemic shunt
HE: Hepatic encephalopathy
MELD: Model of end-stage liver disease
LiMAx: Liver maximum capacity
LT: Liver transplant
PTFE: Polytetrafluoroethylene
AUROC: Area under the receiver operating characteristics
NASH: Non-alcoholic steatohepatitis
HCV: Hepatitis C virus
PBC: Primary biliary cholangitis
AIH: Autoimmune hepatitis
ROC: Receiver operating characteristic
AST: Aspartate aminotransferase
ALT: Alanine aminotransferase
INR: International normalized ratio
NPV: Negative predictive value
PPV: Positive predictive value
